# Supplementary material for: A high-resolution mRNA expression time course of embryonic development in zebrafish
Source: eLife. 2017 Nov 16;6:e30860. doi: 10.7554/eLife.30860 (PMC5690287; doi:10.7554/eLife.30860)
Supplement: Supplementary file 6. [file elife-30860-supp6.zip › biolayout-clusters-files/Cluster181-genes.html]

Cluster181


# Cluster181: Genes

| Ensembl ID | Gene Name | Chr | Start | End | Biotype |
| --- | --- | --- | --- | --- | --- |
| ENSDARG00000092281 | FLNB (1 of many) | 23 | 19679948 | 19726572 | protein\_coding |
| ENSDARG00000015495 | klf3 | 1 | 17586972 | 17922126 | protein\_coding |
| ENSDARG00000094732 | mical3b | 4 | 9723276 | 9763853 | protein\_coding |
| ENSDARG00000023181 | pcp4l1 | 2 | 38012852 | 38017946 | protein\_coding |
| ENSDARG00000063133 | slc4a10a | 11 | 10685769 | 10814329 | protein\_coding |
| ENSDARG00000013730 | slc4a4a | 5 | 45147260 | 45276787 | protein\_coding |
| ENSDARG00000010794 | tfeb | 11 | 22127519 | 22200738 | protein\_coding |
| ENSDARG00000012281 | zgc:65851 | 5 | 13171397 | 13184830 | protein\_coding |
